# Nivolumab plus ipilimumab versus lenvatinib or sorafenib for US and Chinese patients with unresectable hepatocellular carcinoma: a cost-effectiveness analysis

**DOI:** 10.3389/fpubh.2025.1726477

**Published:** 2026-01-12

**Authors:** Qiuji Wu, Yi Qin, Qiu Li

**Affiliations:** 1 Division of Abdominal Tumor Multimodality Treatment, Cancer Center, West China Hospital, Sichuan University, Chengdu, China; 2 Department of Radiation Oncology, Sichuan Clinical Research Center for Cancer, Sichuan Cancer Hospital & Institute, Sichuan Cancer Center, Affiliated Cancer Hospital of University of Electronic Science and Technology of China, Chengdu, China

**Keywords:** cost-effectiveness, dual immunotherapy, first-line therapy, hepatocellular carcinoma, partitioned survival model

## Abstract

**Background:**

The CheckMate 9DW trial showed that nivolumab plus ipilimumab (NIVO + IPI) improves overall survival over lenvatinib or sorafenib (LEN/SOR) in patients with unresectable hepatocellular carcinoma (uHCC). We evaluated the cost-effectiveness of NIVO + IPI versus LEN/SOR from payer perspectives in the United States (US) and China, explicitly aiming to inform reimbursement and price-negotiation decisions in each setting.

**Methods:**

Based on the overall and subgroup data from the CheckMate 9DW trial, we developed a partitioned survival model to compare the clinical outcomes of NIVO + IPI versus LEN/SOR. Costs included drugs, administration, monitoring, adverse event management, and follow-up care in both the US and China. A lifetime horizon (1-month cycles) and 2024 US dollars were applied. The primary outcome was the incremental cost-effectiveness ratio (ICER), expressed as cost per quality-adjusted life year (QALY) gained. Willingness-to-pay (WTP) thresholds were $150,000/QALY (US) and $39,933.67/QALY (China). Model uncertainty was assessed through one-way and probabilistic sensitivity analyses.

**Results:**

In the US, the NIVO + IPI regimen generated an incremental gain of 0.68 QALYs at an additional cost of $86,403.43, resulting in an ICER of $127,063.87 per QALY. In the China subgroup, NIVO + IPI yielded an incremental gain of 0.94 QALYs with an incremental cost of $35,358.47, corresponding to an ICER of $37,615.40 per QALY. Sensitivity analyses indicated that the ICER was mostly influenced by variations in the discount rate and drug prices. Probabilistic sensitivity analysis demonstrated that the probability of NIVO + IPI being cost-effective exceeded 50% at the respective WTP thresholds in both the United States and China.

**Conclusion:**

This study suggests that NIVO + IPI is cost-effective in both the United States and China at current price levels.

## Introduction

Hepatocellular carcinoma (HCC) is a common malignancy worldwide and is associated with high mortality (1, 2). China bears a disproportionate share of the global liver cancer burden, accounting for nearly half of all HCC cases and associated deaths (3, 4). The major etiological factors in China include hepatitis B virus infection, aflatoxin exposure, and cirrhosis (5). In the United States (US), approximately 42,240 new liver cancer cases and 30,090 deaths are projected to occur in 2025. Although the overall incidence of HCC in the US is lower than that in high-incidence regions such as East Asia, the proportion of cases attributable to smoking, obesity, diabetes, and non-alcoholic fatty liver disease has increased in recent years, further intensifying the public health burden (6–8). Patients diagnosed with unresectable HCC (uHCC) have poor outcomes, with median overall survival (OS) generally ranging from 10 to 14 months and population-based 5-year relative survival for distant-stage liver cancer remaining around 3 to 4% (9, 10) (11).

Since sorafenib was established as the standard first-line therapy in 2007, targeted therapies have led to moderate improvements in OS for patients with uHCC (9, 12). However, their clinical benefits are limited by relatively low objective response rates (ORR) and progression-free survival (PFS), and they are frequently associated with adverse events—such as hand-foot syndrome, hypertension, and diarrhea—that may compromise treatment tolerability (13). The REFLECT trial demonstrated that lenvatinib was non-inferior to sorafenib in terms of OS and showed superior ORR and PFS. However, the median OS achieved with lenvatinib remains limited (10).

The application of immune checkpoint inhibitors has opened new avenues for the treatment of advanced HCC. Based on the results of the CheckMate 040 study, the combination of nivolumab and ipilimumab (NIVO + IPI) demonstrated durable responses in patients previously treated with sorafenib, which led to its accelerated approval by the US Food and Drug Administration (FDA) (14, 15). Subsequently, the phase III CheckMate 9DW trial evaluated the efficacy and safety of NIVO + IPI compared with lenvatinib or sorafenib (LEN/SOR) in patients with advanced HCC who had not received prior systemic therapy (16). The study enrolled 668 patients. With a median follow-up of 35.2 months, the median OS was extended to 23.7 months compared to 20.6 months in the control group (hazard ratio [HR] = 0.79; 95% confidence interval [CI] 0.65–0.96; *p* = 0.018). Survival rates at 24 and 36 months were also significantly higher in the NIVO + IPI group compared to the LEN/SOR group (16). These results prompted the approval of nivolumab plus ipilimumab by the FDA, NMPA and NCCN as a first-line treatment option for patients with uHCC (17–19).

Although the NIVO + IPI regimen demonstrates significant clinical advantages as a first-line treatment, its high costs and associated healthcare resource consumption present substantial challenges to current healthcare payment systems. In the US, where market-based pricing and a multi-tiered insurance system prevail, there is growing scrutiny of novel drug pricing and incremental cost-effectiveness ratios (ICERs). In China, substantial price reductions are achieved through National Reimbursement Drug List negotiations. However, pressure on the healthcare fund persists, and health technology assessment criteria for cost-effectiveness evaluations are becoming more stringent. Accordingly, we used data from CheckMate 9DW to develop a model evaluating the cost-effectiveness of first-line NIVO + IPI versus LEN/SOR from the perspectives of the US and Chinese health care systems. This analysis will provide quantitative evidence to support clinical decision-making, reimbursement negotiations, and health policy development.

## Materials and methods

This study aims to assess the cost-effectiveness of dual immunotherapy (nivolumab plus ipilimumab) compared to single-agent targeted therapies (sorafenib or lenvatinib) as a first-line treatment for uHCC, based on data from the CheckMate 9DW trial (16). Specifically, the US analysis was informed by the overall CheckMate 9DW population (16), whereas the Chinese analysis used overall survival data from the Chinese subgroup of CheckMate 9DW (20). A model was constructed to perform the analysis from the perspectives of both the United States and Chinese healthcare systems. The evaluation adheres to the Consolidated Health Economic Evaluation Reporting Standards 2022 guidelines (Supplementary Table S1).

### Participants and procedures

The target population of the model was derived from the inclusion criteria of the CheckMate 9DW trial, which included patients aged ≥18 years with treatment-naïve, uHCC, a Child-Pugh score of 5–6, an Eastern Cooperative Oncology Group performance status of 0–1, and at least one measurable lesion according to RECIST 1.1 criteria. A total of 668 patients were randomized, with 335 assigned to the dual immunotherapy group and 333 to the single-agent targeted therapy group (16). In the Chinese subgroup, 98 patients were assigned to the NIVO + IPI group, and 110 patients were assigned to the LEN/SOR group (20). The model assumed the target population was identical to that of the CheckMate 9DW trial and assigned patients 1:1 to either the NIVO + IPI group or the LEN/SOR group. In the NIVO + IPI group, patients received intravenous nivolumab at 1 mg/kg and ipilimumab at 3 mg/kg every 3 weeks for up to four doses, followed by maintenance nivolumab monotherapy at 480 mg every 4 weeks. Patients in the LEN/SOR group received either oral lenvatinib at 8 mg daily (<60 kg body weight) or 12 mg daily (≥60 kg), or oral sorafenib at 400 mg twice daily (16). The analysis was performed from the perspectives of both the US and Chinese healthcare systems.

### Model structure and survival

This study employed a state-transition model to simulate the progression of patients with uHCC following first-line treatment with either dual immunotherapy or single-agent targeted therapy, while simultaneously capturing associated costs and utility values. The model consisted of four distinct health states: PFS, second PFS (PFS2), progressive disease (PD), and death. Death was modeled as an absorbing state, and all transitions between health states were assumed to be irreversible (Supplementary Figure S1). The PFS state represented the clinical phase in which patients receiving first-line therapy exhibit no disease progression. The PFS2 is defined as the period from the first objective disease progression to the second objective progression or death. The PD state indicates further disease deterioration following second-line treatment. All patients entered the model in the PFS state, receiving either dual immunotherapy or single-agent targeted therapy as first-line treatment until disease progression or intolerable toxicity occurred. Patients who progressed during first-line therapy transitioned to the PFS2 state to receive second-line anti-cancer treatment. Patients in the PFS2 state could further transition to the PD state upon experiencing a second progression. Ultimately, patients in any state could transition directly to death. The model was constructed using a one-month cycle length and a 20-year time horizon to reflect lifetime clinical and economic outcomes.

Transition probabilities were primarily estimated from the published Kaplan–Meier (KM) curves for OS and PFS from the CheckMate 9DW trial. To accurately reflect regional prognostic differences, the transition probabilities for the US analysis were derived from the intention-to-treat (ITT) population, whereas the analysis from the Chinese perspective utilized OS data specifically from the Chinese subgroup. To extrapolate survival beyond the trial follow-up period, WebPlotDigitizer®^1^ was employed to digitize the published PFS and OS curves by extracting time–survival probability coordinates and the number-at-risk data (21). Individual patient data (IPD) were reconstructed using R packages such as “survHE” and “IPDfromKM,” following the methodology developed by Baio et al. (22), enabling subsequent parametric survival modeling. The reconstructed IPDs were fitted using several standard parametric distributions, including exponential, Weibull, Gompertz, log-normal, and log-logistic functions. Model parameters were estimated using maximum likelihood estimation, and model fit was evaluated using the Akaike information criterion and Bayesian information criterion. Given the potential structural uncertainty in long-term survival projections from a single parametric model, Bayesian model averaging was employed to integrate predictions from multiple models through weighted averaging based on AIC values. This method aimed to mitigate biases arising from the limitations of individual models (23). Finally, the PFS and OS survival functions for the dual immunotherapy and single-agent targeted therapy groups were calculated based on Bayesian model average (BMA) weights (Supplementary Table S2) (23), and the goodness-of-fit between the reconstructed and original KM curves was visually assessed (Supplementary Figures S2–S5).

### Costs and utilities

The cost analysis included only direct medical costs, including drug, administration, management of adverse events (AE), follow-up, second-line therapy, best supportive care, and end-of-life care. Drug costs were calculated based on the dosing regimens used in the trial and wholesale acquisition costs (WAC) sourced from Red Book® or publicly available databases (24, 25). Administration costs were obtained from the 2023 Medicare Physician Fee Schedule or estimated values (Supplementary Table S3). Costs associated with AE management were limited to grade 3 or higher AEs that showed significant between-group differences in the CheckMate 9DW study. AE unit costs were derived from published literature or estimated, with AE incidence rates, unit costs, and sources detailed in Supplementary Table S4. The costs of subsequent systemic therapies were calculated as weighted mean values, obtained by multiplying the proportion of patients receiving each category of subsequent regimen in the CheckMate 9DW trial by the corresponding regimen-specific cost per 1-month cycle and summing across all categories, and aligned with treatment guidelines specific to national healthcare contexts (Supplementary Table S5). Patients who did not receive subsequent antitumor therapy were assumed to receive best supportive care. End-of-life care costs were modeled as a one-time expense. The costs of follow-up, best supportive care, and palliative care were obtained from published literature (Supplementary Table S6).

The estimation of quality-adjusted life years (QALYs) was based on health state utilities (HSUs), calculating HSU values for progression-free and progressive disease states separately while assuming consistent HSU values across treatment groups. Since the CheckMate 9DW trial did not provide quality-of-life data, we obtained health utility values from previously published literature. The utility values were 0.84 for PFS, 0.76 for PFS2, and 0.68 for PD, with 0 assigned to the death state (Supplementary Tables S7,S8). These values were applied to both treatment group (26–28). Additionally, disutility associated with treatment-related adverse events was incorporated from published sources, with the assumption that such events occurred only during the first cycle.

### Cost-effectiveness analysis

The base-case analysis assessed the cost-effectiveness of nivolumab plus ipilimumab compared with lenvatinib or sorafenib as first-line treatments for advanced HCC by comparing ICERs and QALYs. Given the absence of an officially defined willingness-to-pay (WTP) threshold in the United States, a benchmark of $150,000 per QALY—commonly recommended by ICER—was used for interpretation. In contrast, China’s WTP threshold was defined as three times the national per capita gross domestic product (GDP), amounting to $39,933.67 per QALY (29). Costs and utility values were discounted at annual rates of 3% in the United States and 5% in China (30, 31). The primary outcome of the model was the ICER, defined as the incremental cost per QALY gained, expressed in 2024 US dollars. Model construction and analysis were conducted using TreeAge Pro™ software.

### Sensitivity, threshold, and scenario analyses

To assess the impact of parameter uncertainty on model outcomes, a series of sensitivity analyses were conducted. In the one-way sensitivity analyses, individual parameters were varied by ±20% from their baseline values or within their respective 95% confidence intervals, and the results were illustrated using tornado diagrams. A two-way sensitivity analysis was also performed to examine the joint effect of varying the prices of nivolumab and ipilimumab. For the probabilistic sensitivity analysis (PSA), 1,000 Monte Carlo simulations were performed, with all parameters varied simultaneously. Gamma distributions were applied to cost-related parameters, including drug acquisition, monitoring, and AE management—while beta distributions were used for health utility values associated with PFS and PD. The outcomes of the PSA were presented using cost-effectiveness acceptability curves and scatter plot.

In addition, threshold and scenario analyses were conducted to assess the impact of key parameters on the ICER and evaluate the robustness of the model outcomes. The threshold analysis involved systematically varying the prices of nivolumab in combination with ipilimumab to determine the price points at which the regimen would achieve cost-effectiveness under different WTP thresholds in both the United States and China. In the US, WTP thresholds of $150,000, $100,000, and $50,000 per QALY were applied. In China, WTP thresholds were set at three times the national per capita GDP, as well as three times the per capita GDP of the provinces with the lowest ($22,031.53) and highest income levels ($95,160.78). The scenario analysis examined a range of alternative assumptions, including altering PFS, and shortening the model time horizon from lifetime to 4, 5, 10 years.

## Results

### Base case results

The results of the base-case cost-effectiveness analysis are summarized in Table 1. From the perspective of the US healthcare system, the NIVO + IPI regimen was associated with a lifetime cost of $804,965.13, compared to $718,561.70 for the LEN/SOR group, resulting in an incremental cost of $86,403.43. In terms of effectiveness, the NIVO + IPI group gained an additional 0.68 QALYs compared to the control group. Consequently, the ICER was estimated at $127,063.87 per QALY. Given that this ICER falls below the WTP threshold of $150,000 per QALY, the NIVO + IPI regimen is considered a cost-effective treatment option in the US setting. In the analysis conducted from the Chinese healthcare perspective, the total lifetime cost for NIVO + IPI was $109,160.95, exceeding the cost of LEN/SOR ($73,802.48) by $35,358.47. The corresponding ICER was calculated at $37,615.40 per QALY. This value is below China’s WTP threshold of $39,933.67 per QALY, indicating that NIVO + IPI is also cost-effective at current prices within the Chinese setting. Notably, when the analysis was restricted to consider only the gains from PFS, the ICERs increased substantially, reaching $1,008,089.36 per QALY in the US and $129,851.46 per QALY in China. This marked discrepancy underscores that the OS benefit is the predominant driver of the regimen’s cost-effectiveness profile.

**Table 1:** Results of base-case analysis.

Strategy		Costs ($)	Incremental costs ($)	Effectiveness (LY)	Effectiveness (QALY)	Incremental effectiveness	ICER ($)
Overall survival							
US	NIVO + IPI	804,965.13	86,403.43	4.15	3.20	0.68	127,063.87
	LEN/SOR	718,561.70		3.28	2.52		
China	NIVO + IPI	109,160.95	35,358.47	4.23	3.32	0.94	37,615.40
	LEN/SOR	73,802.48		3.07	2.38		
Only progression-free survival							
US	NIVO + IPI	670,241.15	393,154.85	1.53	1.26	0.39	1,008,089.36
	LEN/SOR	277,086.3		1.04	0.87		
China	NIVO + IPI	100,062.23	81,806.42	1.53	1.49	0.63	129,851.46
	LEN/SOR	18,255.81		1.04	0.86		

NIVO + IPI, nivolumab plus ipilimumab; LEN/SOR, lenvatinib or sorafenib; LY, life year; QALY, quality adjusted life-year; ICER, incremental cost-effectiveness ratio; US, United States.

### Sensitivity analyses

**Figure 1:**
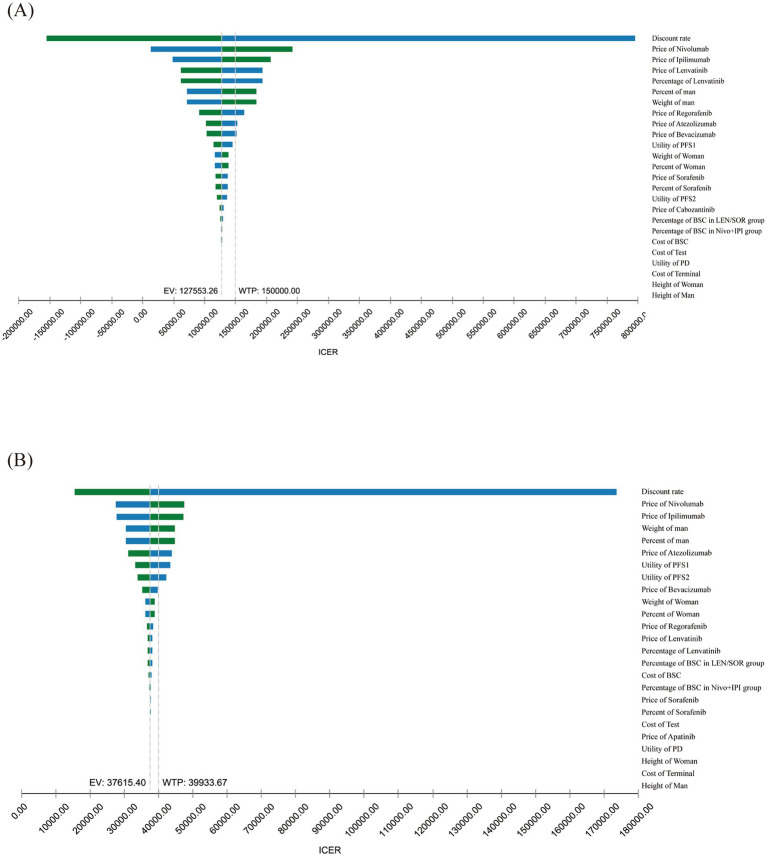
Results of one-way sensitivity analyses. One-way sensitivity analyses for nivolumab plus ipilimumab strategy compared with lenvatinib or sorafenib strategy in the United States **(A)** and China **(B)**. PD, progressive disease; PFS, progression-free survival; BSC, best supportive care.

**Figure 2:**
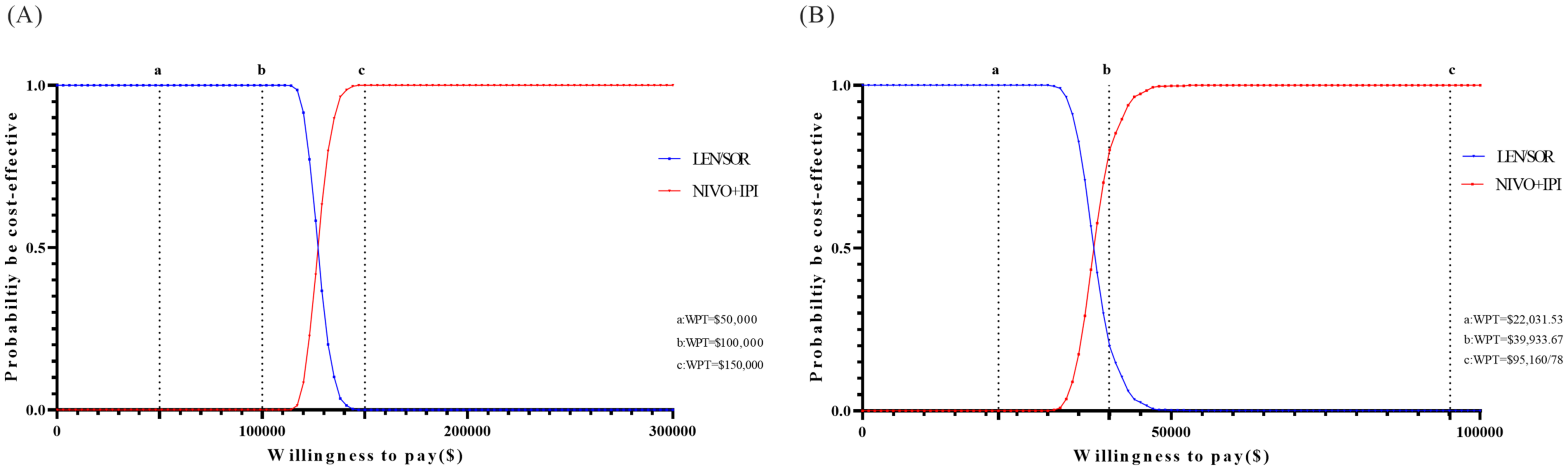
Results of probabilistic sensitivity analyses. Cost-effectiveness acceptability curves for nivolumab plus ipilimumab strategy compared with lenvatinib or sorafenib strategy in the United States **(A)** and China **(B)**.

To evaluate the impact of parameter uncertainty on cost-effective outcomes, a one-way sensitivity analysis was conducted. In the United States, the results indicated that the discount rate had the most significant influence on the ICER, followed by the prices of nivolumab and ipilimumab. The price of lenvatinib and its utilization rate also had a notable impact. In contrast, factors such as patient body weight, gender distribution, and the cost of subsequent treatments demonstrated relatively limited effects. Overall, the sensitivity analysis indicated that the cost-effectiveness of the NIVO + IPI regimen is relatively robust within the US healthcare context. Nevertheless, fluctuations in drug pricing may cause the ICER to exceed the WTP threshold. Similarly, in China, the discount rate exerts the greatest influence on the ICER, followed by the prices of nivolumab and ipilimumab, ranking second and third, respectively (Figures 1A,B). Other parameters, such as the proportion of male patients, average body weight, and the price of lenvatinib, demonstrate relatively limited impact. PSA was performed using Monte Carlo simulations to assess the cost-effectiveness probability of the NIVO + IPI regimen compared to lenvatinib or sorafenib for the treatment of uHCC in the United States and China. In the United States, the probability was 0% at a WTP threshold of $50,000 and $100,000 per QALY, and reached 100% at $150,000/QALY. In China, the probability of NIVO + IPI being cost-effective was 0% at a WTP threshold of $22,031.53 per QALY, increased to 81.7% at $39,933.67 per QALY, and reached 100% at $95,160.78 per QALY, highlighting the regimen’s increasing economic advantage at higher WTP levels (Figures 2A,B). The scatter plot from the probabilistic sensitivity analysis illustrates the distribution of cost-effectiveness outcomes across varying WTP thresholds (Supplementary Figures S6A,B).

### Threshold analyses

**Figure 3:**
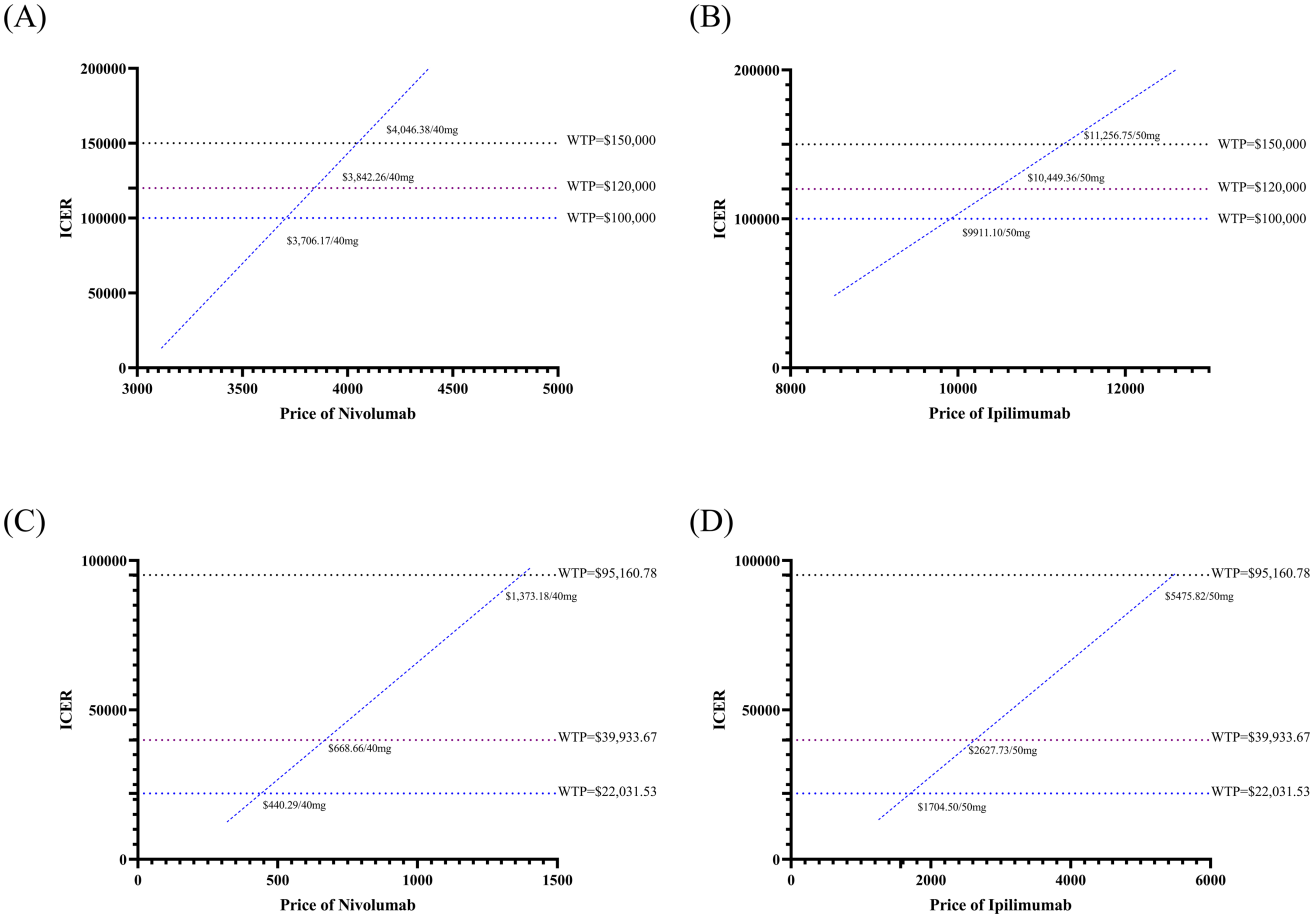
Results of price simulation. **(A)** Price simulation of Nivolumab in the United States; **(B)** Price simulation of Ipilimumab in the United States; **(C)** Price simulation of nivolumab in the China states; **(D)** Price simulation of ipilimumab in China; WTP, willingness-to-pay.

At current US pricing levels, the ICER of the NIVO + IPI regimen remained below the commonly cited WTP threshold of US$150,000 per QALY. In exploration price-threshold analyses, we examined how changes in drug unit prices would impact the economic robustness of the regimen at a lower WTP threshold of US$100,000 per QALY. Under this threshold, relatively small reductions in the unit price of nivolumab (≈5%) or ipilimumab (≈7%) were sufficient to maintain the ICER below US$100,000 per QALY. When applying simultaneous price reductions to both agents, a coordinated decrease of around 3% in the unit price of each drug achieved an ICER below this threshold (Figures 3A,B, Supplementary Figures S7A–C). In China, at a WTP threshold of US$39,933.67 per QALY, the regimen remained cost-effective within a narrow range of potential price increases (approximately 5% for each agent; Figures 3C,D). When adopting a lower WTP threshold of US$22,031.53 per QALY, simultaneous price reductions of about 15–16% in the unit price of both drugs were required for the ICER to fall below the threshold (Supplementary Figures S7D,F). Additional price-threshold analyses for monotherapy and combination regimens across alternative WTP levels in both the United States and China are presented in Supplementary Figure S7.

### Scenario analyses

Multiple scenario simulations were conducted to assess the robustness of the base-case conclusions. First, regarding model assumptions, the utilization of investigator-assessed PFS curves resulted in a significant decrease in the ICER. Conversely, the exclusion of disutilities associated with AEs had a negligible impact on the outcomes. Notably, shortening the model time horizon (e.g., to 10, 5, or 4 years) led to a substantial increase in ICERs across all settings, underscoring the critical contribution of long-term survival benefits to the regimen’s economic value (Table 2). In addition, to account for the cost disparity between comparator agents, we performed separate cost-effectiveness analyses for NIVO + IPI against specific comparators (lenvatinib or sorafenib monotherapy) utilizing the overall trial population for both the US and Chinese settings. When compared specifically with lenvatinib, the ICER for NIVO + IPI was $201,266.26 per QALY in the US and $42,641.28 per QALY in China. In contrast, when compared with sorafenib, the corresponding ICERs were $179,934.87 per QALY (US) and $27,984.19 per QALY (China) (Supplementary Table S9). An analysis using the overall population yielded results consistent with the primary Chinese subgroup analysis, reinforcing model robustness (Supplementary Table S10).

**Table 2:** Scenario analysis results.

Strategy	US					CN				
	Costs ($)	Incremental costs ($)	Effectiveness (QALY)	Incremental effectiveness	ICER ($)	Costs ($)	Incremental costs ($)	Effectiveness (QALY)	Incremental effectiveness	ICER ($)
Scenario 1: PFS investigator										
NIVO + IPI	824,585.97	114,710.87	3.86	1.39	82,739.12	108,278.32	34,852.17	4.00	1.68	20,745.34
LEN/SOR	709,875.11		2.47			73,426.16		2.32		
Scenario 2: exclude AE disutilities										
NIVO + IPI	804,965.13	86,403.43	3.22	0.69	124,748.49	109,160.95	35,358.47	3.34	0.96	36,831.74
LEN/SOR	718,561.70		2.53			73,802.48		2.38		
Scenario3: time horizon 4 years										
NIVO + IPI	769769.88	149277.68	2.22	0.20	746,388.38	115,774.36	53,085.06	2.35	0.33	162,977.03
LEN/SOR	620492.20		2.02			62,689.30		2.02		
Scenario 4: time horizon 5 years										
NIVO + IPI	787,952.60	131,851.71	2.49	0.30	439,505.70	117,415.92	50,325.03	2.69	0.52	97,176.97
LEN/SOR	656,100.89		2.19			67,090.89		2.17		
Scenario 5: time horizon 10 years										
NIVO + IPI	804,410.42	95,079.62	3.05	0.61	155,446.36	118,585.49	45,116.30	3.30	0.93	48,512.15
LEN/SOR	709,330.80		2.44			73,469.19		2.37		

NIVO+IPI, nivolumab plus ipilimumab; QALY, quality adjusted life-year; ICER, incremental cost-effectiveness ratio; US, United States.

## Discussion

This study used a model based on data from the CheckMate 9DW trial to evaluate the cost-effectiveness of NIVO + IPI versus LEN/SOR as first-line treatment for patients with uHCC. In the United States, the ICER of NIVO + IPI compared with LEN/SOR was US$127,063.87 per QALY, which is below the WTP threshold of US$150,000 per QALY, indicating that NIVO + IPI is cost-effective in this setting. Similarly, in China, the ICER of NIVO + IPI versus LEN/SOR was US$37,615.40 per QALY, slightly below the WTP threshold of US$39,933.67 per QALY, suggesting that NIVO + IPI is also cost-effective at current prices. In the United States, the favorable cost-effectiveness of NIVO + IPI is largely attributable to the relatively high WTP threshold, which reflects the greater fiscal capacity and higher societal willingness to pay for innovative oncologic therapies. The clinical benefits of NIVO + IPI further reinforce its economic value in this context. By contrast, China applies a substantially lower WTP threshold, and drug prices remain relatively high, meaning that the cost-effectiveness of NIVO + IPI compared with LEN/SOR in the Chinese base-case analysis is more sensitive to price assumptions. Sensitivity analyses showed that in both the United States and China, price changes of approximately ±20% for NIVO and IPI could result in NIVO + IPI no longer being cost-effective relative to LEN/SOR.

In April 2025, the US FDA approved NIVO + IPI as a first-line treatment for patients with unresectable or metastatic HCC, further confirming its clinical value (17). Moreover, the high willingness to pay for innovative cancer therapies within the US healthcare system supports the broader adoption of NIVO + IPI and may promote its elevation in clinical guidelines, such as updates to NCCN recommendations (18). In the long term, this could incentivize pharmaceutical companies to further invest in immunotherapy research for HCC and to explore more effective combination strategies.

In China, although the regimen is cost-effective at current prices, the ICER approaches the upper limit of the WTP threshold. This high absolute value suggests that inclusion in the National Reimbursement Drug List (NRDL) may face challenges, potentially limiting patient access. However, our scenario analysis reveals that cost-effectiveness is highly dependent on the choice of comparator. In the Chinese setting, NIVO + IPI is cost-effective compared with sorafenib monotherapy (ICER: $27,984/QALY) but not compared with lenvatinib monotherapy (ICER: $42,641/QALY). This pattern indicates that for patients where sorafenib is the relevant clinical alternative, NIVO + IPI offers acceptable economic value even at current prices; however, displacing lenvatinib as the standard of care would necessitate price concessions. This distinction provides a rationale for policymakers to prioritize the therapy for specific patient subgroups. To improve accessibility, the National Healthcare Security Administration (NHSA) could leverage centralized procurement and price negotiations to lower drug costs, drawing on experiences with other immune checkpoint inhibitors where price reductions have exceeded 50% (32). While the commercial feasibility of such drastic reductions for the NIVO + IPI combination remains uncertain, alternative mechanisms-such as patient assistance programs or tiered reimbursement policies-could be implemented to subsidize treatment for economically disadvantaged populations, thereby balancing clinical benefit with economic burden. Furthermore, although a formal provincial-level analysis was not conducted, significant regional disparities in per capita GDP suggest that the WTP threshold in economically developed provinces may exceed the national average, potentially enhancing the cost-effectiveness of NIVO + IPI in these regions. Future research incorporating provincial cost and epidemiological data is warranted to quantify these regional variations.

The divergent findings between the two countries further underscore the necessity of context-specific healthcare policies. In the United States, a market-oriented system with higher WTP thresholds facilitates the rapid adoption of innovative therapies. In contrast, China’s emphasis on cost containment and equitable access necessitates more rigorous economic evaluations. Consequently, policymakers should formulate differentiated reimbursement and pricing strategies that reflect national economic conditions to ensure patients have access to high-value treatments.

Several economic evaluations have examined immune checkpoint inhibitor–based combinations as first-line treatment for uHCC in different settings. Analyses of tremelimumab plus durvalumab generally suggest that this regimen can be cost-effective in the United States when compared with sorafenib (33–36). In contrast, cost-effectiveness studies of atezolizumab plus bevacizumab versus sorafenib conducted from US and Chinese payer perspectives have reported ICERs that exceed conventional WTP thresholds at current prices in both countries and have therefore concluded that substantial price reductions would be required to render this combination economically attractive (37, 38). Within this landscape, our findings for nivolumab plus ipilimumab present a distinct and favorable profile: the regimen yields ICERs below the commonly accepted WTP thresholds in both the United States and China. Taken together, these results indicate that while specific conclusions differ across regimens, the economic value of ICI-based combinations for uHCC is highly sensitive to drug prices and to country-specific willingness-to-pay thresholds.

Our study offers several strengths. First, to our knowledge, it represents the first cost-effectiveness assessment of NIVO + IPI as a first-line therapy for uHCC. This analysis is poised to inform healthcare policy development and clinical decision-making in both the United States and China. Second, leveraging direct comparisons from the CheckMate 9DW trial between NIVO + IPI and TKIs, our study incorporated the most recent survival data available from that trial for cost-effectiveness modeling. Third, the diverse demographic composition of the CheckMate 9DW trial—comprising 43% of participants from Europe or North America and 42% from Asia—allowed for region-specific modeling considerations to minimize potential bias. This stratification ensures that the cost-effectiveness results are highly relevant and generalizable to the specific patient populations in both the United States and China. Nevertheless, this study has certain limitations. First, reliance on the CheckMate 9DW trial data may not fully capture the heterogeneity of real-world patients, such as those with multiple comorbidities or suboptimal adherence. Second, in the absence of long-term post-follow-up survival data, our projections were based on survival models, which could introduce model bias. To mitigate this, we enhanced the accuracy of the extrapolation by selecting the optimal parametric distribution from multiple candidate survival functions. Third, cost estimates inherently carry uncertainty, as healthcare expenditures vary across regions and institutions, potentially affecting the generalizability of our results. Furthermore, the WTP thresholds for both the United States and China were derived from algorithms prevalent in current academic research, which may not precisely reflect societal preferences for cancer treatment; we addressed these issues through comprehensive sensitivity and threshold analyses under varying scenarios. Finally, due to scarce data on subsequent anticancer therapies, assumptions regarding post-progression treatments based on CheckMate 9DW and NCCN guidelines may not perfectly mirror clinical practice.

## Conclusion

This study demonstrates that NIVO + IPI represents a cost-effective first-line treatment option for uHCC in both the United States and China under current pricing structures. Clinically, the significant survival benefit associated with dual immunotherapy substantiates its status as a preferred therapeutic strategy. From a policy perspective, the favorable economic profile in the United States supports the prioritization of reimbursement coverage. In China, while the regimen meets cost-effectiveness criteria based on population-specific survival data, ensuring broader accessibility and economic robustness through strategic price negotiations or multi-tiered subsidy mechanisms remains essential.

## Data Availability

The original contributions presented in the study are included in the article/Supplementary material, further inquiries can be directed to the corresponding author.

## Footnotes

1
https://automeris.io/WebPlotDigitizer
