# Supplementary material for: Nivolumab plus ipilimumab versus lenvatinib or sorafenib for US and Chinese patients with unresectable hepatocellular carcinoma: a cost-effectiveness analysis
Source: Front Public Health. 2026 Jan 12;13:1726477. doi: 10.3389/fpubh.2025.1726477 (PMC12832771; doi:10.3389/fpubh.2025.1726477)
Supplement: Supplementary file 1 [file Supplementary_file_1.docx]

**Nivolumab Plus Ipilimumab versus lenvatinib or sorafenib for US and Chinese Patients with unresectable hepatocellular carcinoma: A cost-effectiveness analysis**

**Figure S1 Model diagrams.**

**Figure S2 Survival plots showing the goodness-of-fit of OS**

**Figure S3 Survival plots showing the goodness-of-fit of PFS**

**Figure S4 Survival plots showing the goodness-of-fit of PFS2**

**Figure S5 Scatter plots for probabilistic sensitivity analysis.**

**Figure S6 Two-way Sensitivity analysis of Nivolumab plus ipilimumab**

**Table S1 CHEERS 2022 Checklist**

**Table S2 Model choice for all survival data**

**Table S3 Drug acquisition unit costs in the model**

**Table S4 Adverse events occurrences, unit costs of AE management.**

**Table S5 Subsequent anti-cancer therapies for patients in the Nivolumab plus ipilimumab and Lenvatinib or sorafenib groups**

**Table S6 Variables applied in the economic model**

**Table S7 Health state utility values**

**Table S8 Disutility of AE**

# **Figure S1 Model diagrams.**


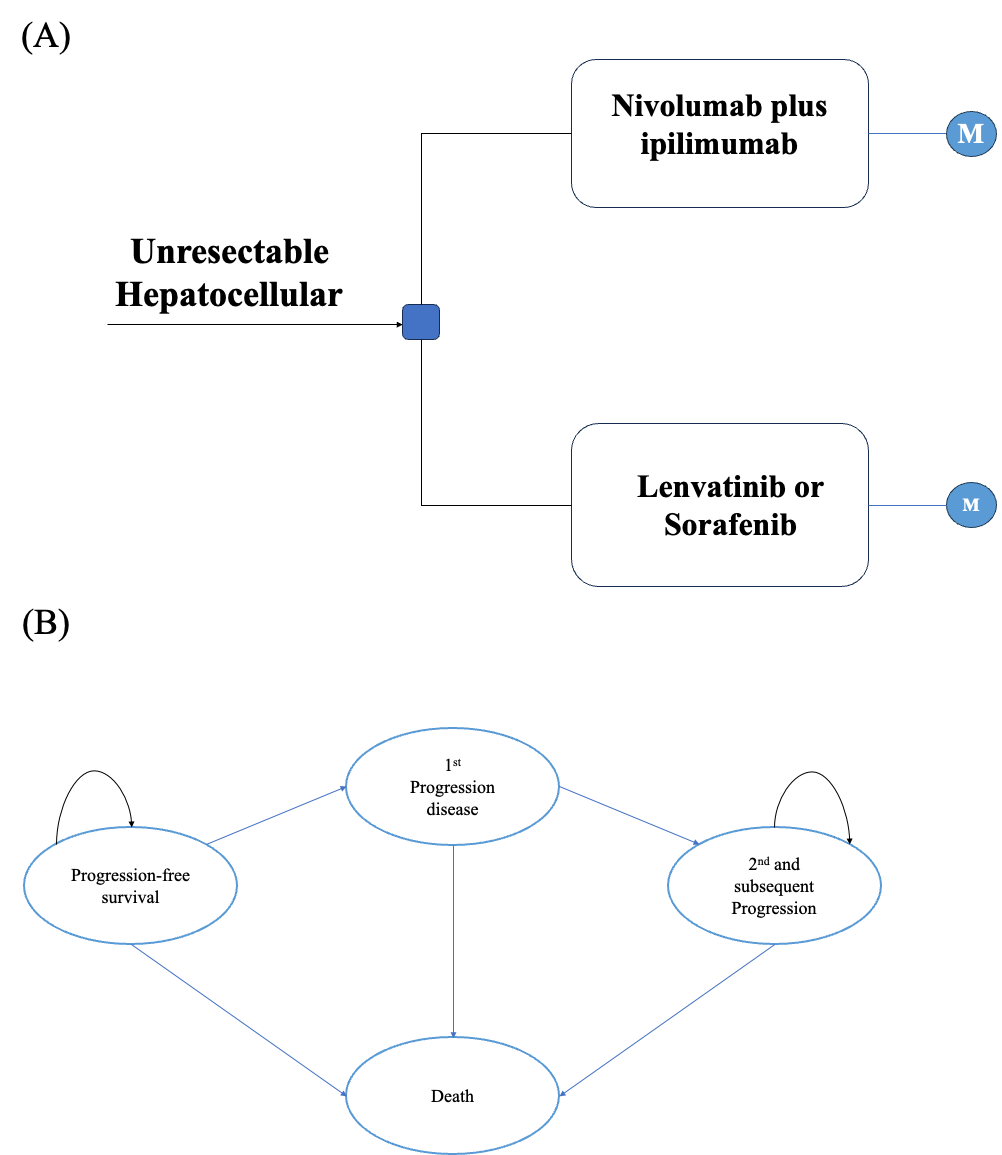


(A) Treatment pathways and decision tree; (B) Simplified Markov Model.

# **Figure S2 Survival plots showing the goodness-of-fit of OS in US.**


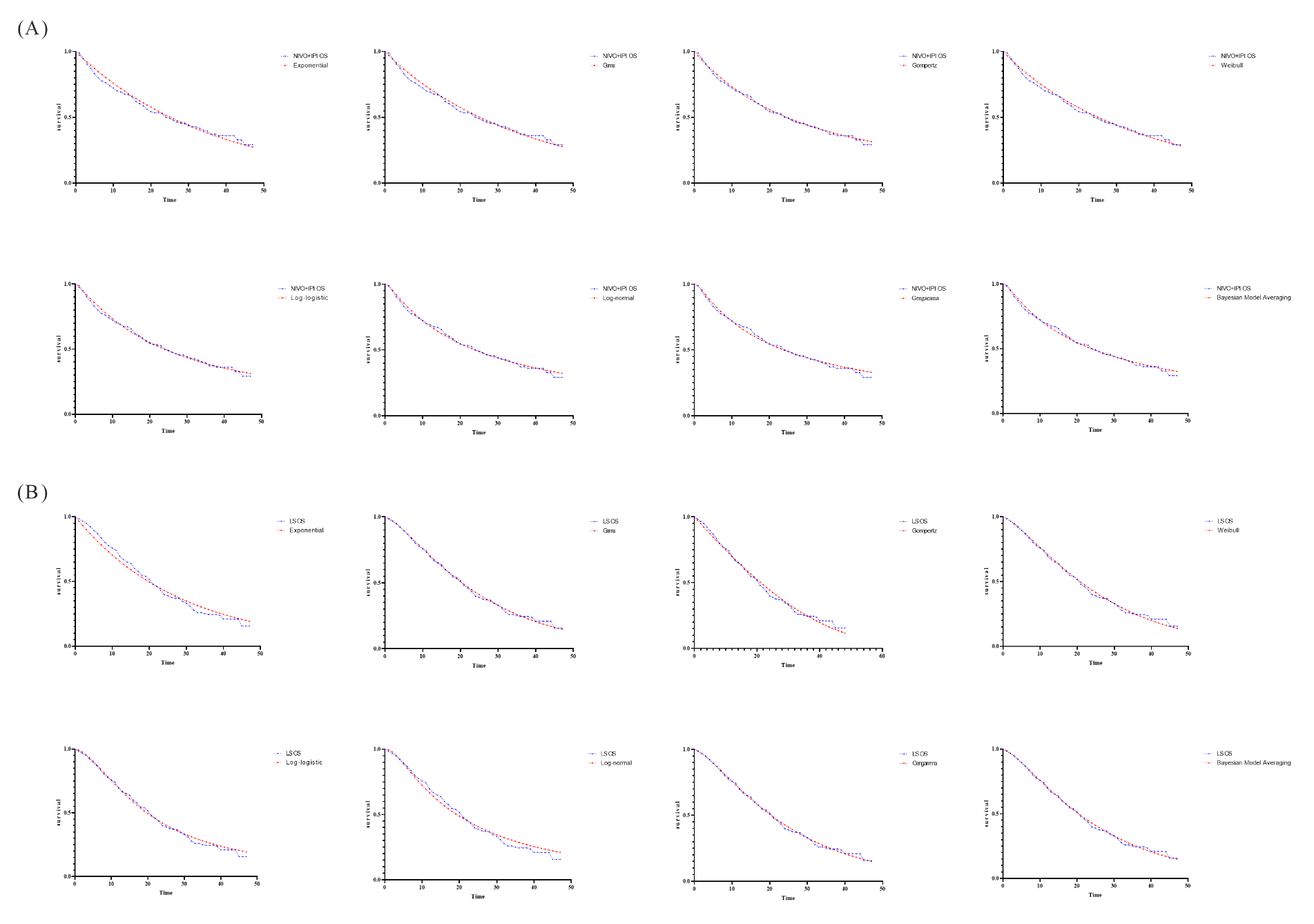


(A) Survival plots for goodness-of-fit of parametric survival models for K-M curve of OS in patients with Nivolumab plus ipilimumab,(B) Survival plots for goodness-of-fit of parametric survival models for K-M curve of OS in patients with Lenvatinib or sorafenib. NIVO+IPI Nivolumab plus ipilimumab,LS, Lenvatinib or sorafenib.

# **Figure S3 Survival plots** **showing the goodness-of-fit of OS in China.**


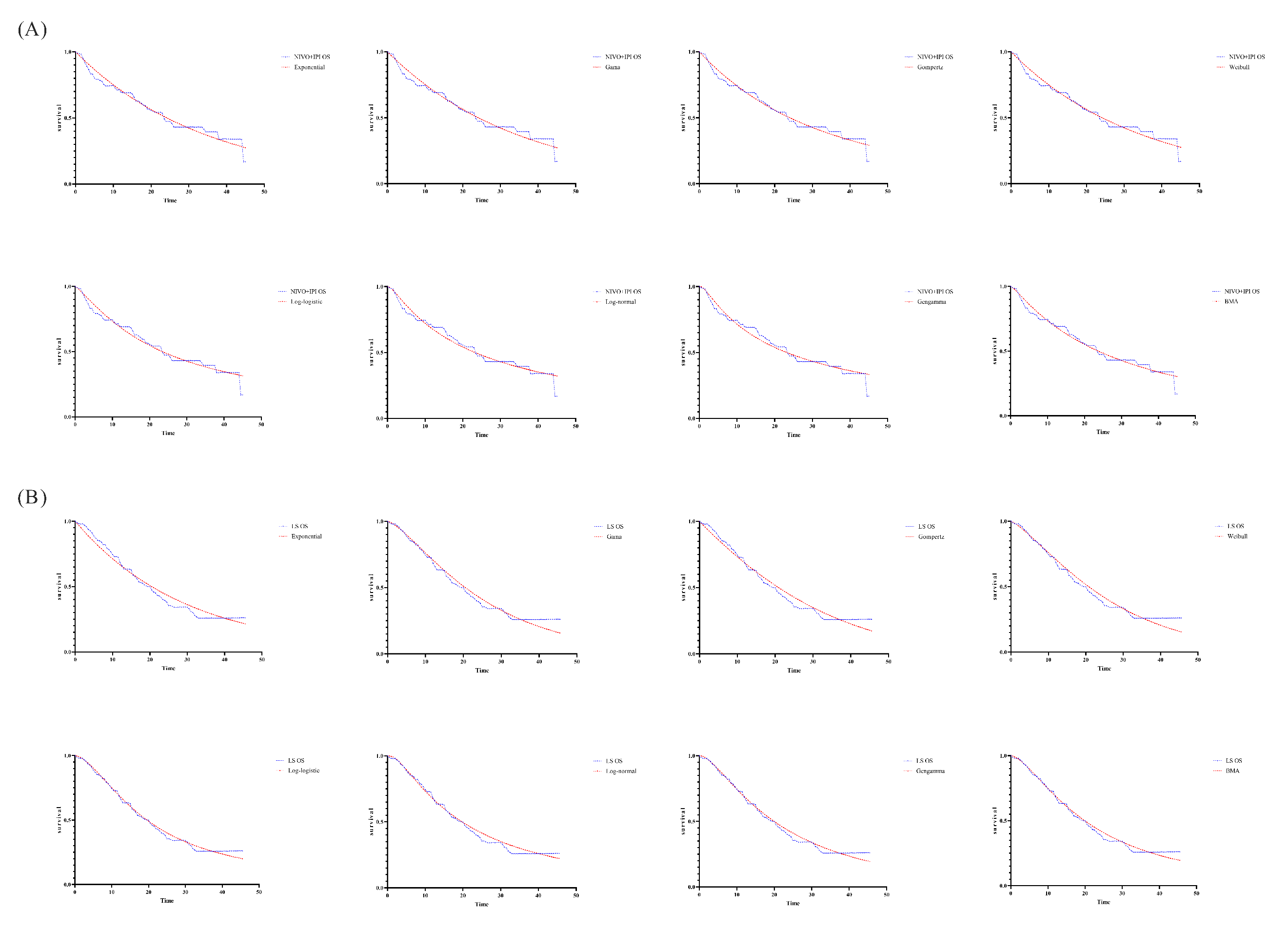


(A) Survival plots for goodness-of-fit of parametric survival models for K-M curve of OS in patients with Nivolumab plus ipilimumab,(B) Survival plots for goodness-of-fit of parametric survival models for K-M curve of OS in patients with Lenvatinib or sorafenib. NIVO+IPI Nivolumab plus ipilimumab,LS, Lenvatinib or sorafenib.

# **Figure S4 Survival plots showing the goodness-of-fit of PFS.**


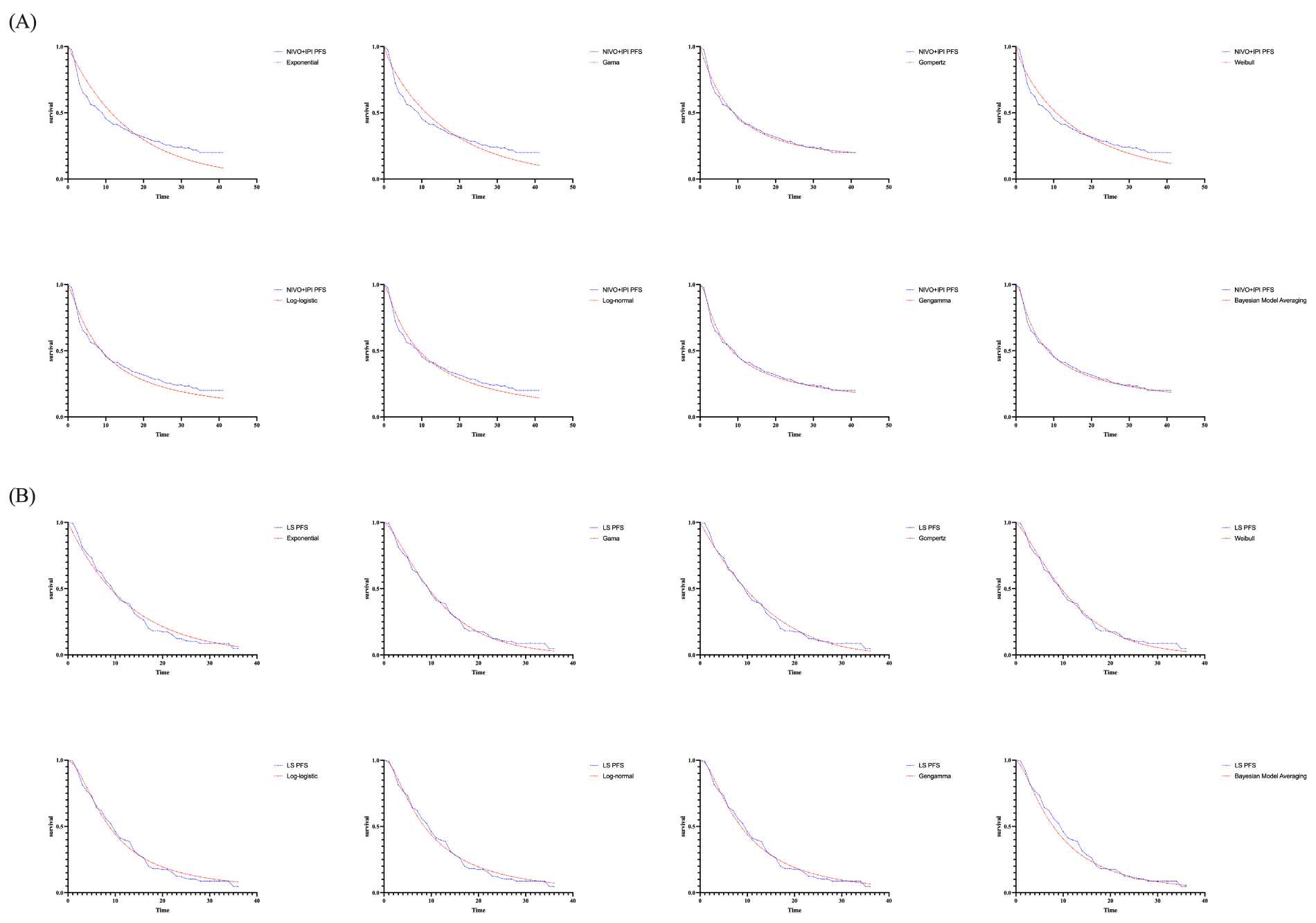
(A) Survival plots for goodness-of-fit of parametric survival models for K-M curve of PFS in patients with Nivolumab plus ipilimumab,(B) Survival plots for goodness-of-fit of parametric survival models for K-M curve of PFS in patients with Lenvatinib or sorafenib. (C) Survival plots for goodness-of-fit of parametric survival models for K-M curve of PFS2 in patients with Nivolumab plus ipilimumab,(D) Survival plots for goodness-of-fit of parametric survival models for K-M curve of PFS2 in patients with Lenvatinib or sorafenib. NIVO+IPI Nivolumab plus ipilimumab,LS, Lenvatinib or sorafenib.

# **Figure S5 Survival plots showing the goodness-of-fit of PFS2.**


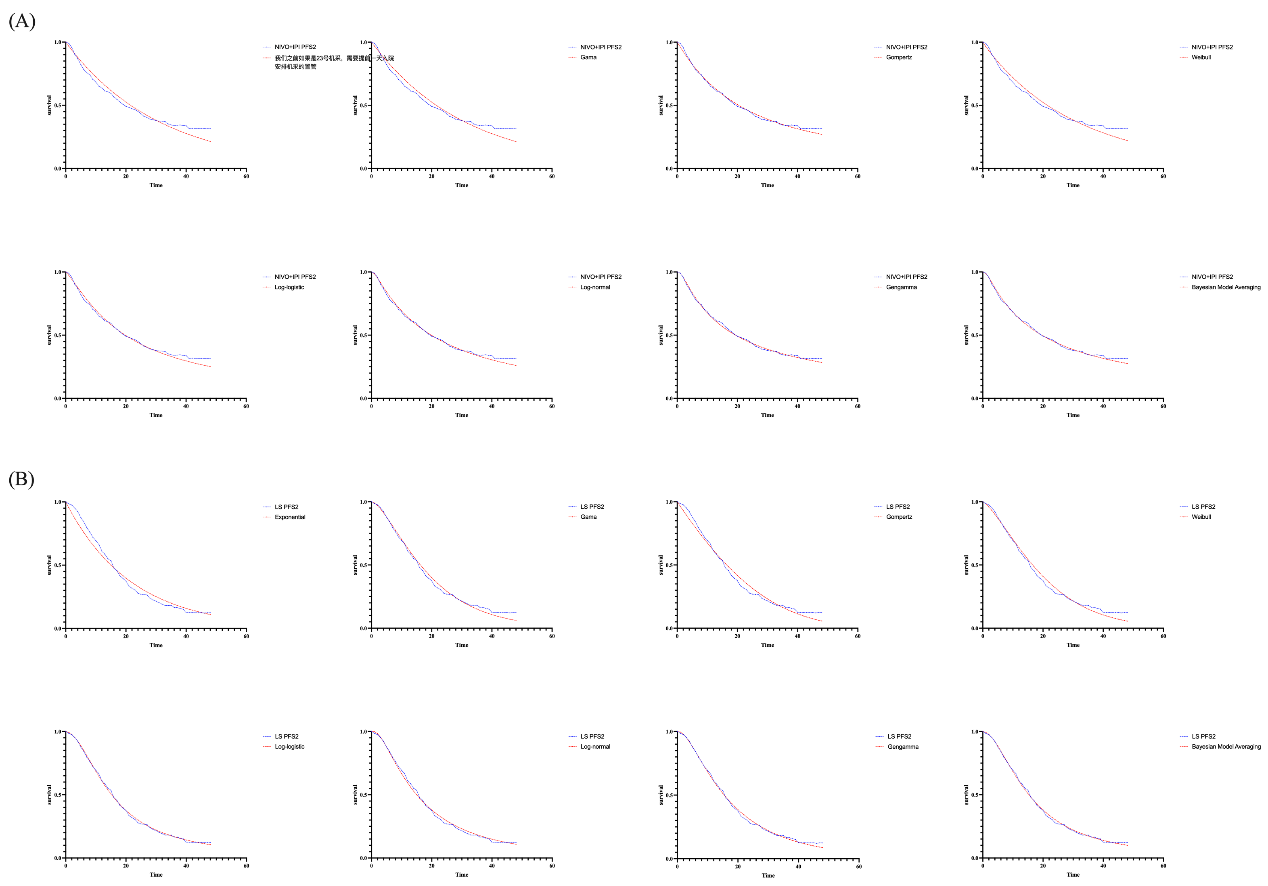
(A) Survival plots for goodness-of-fit of parametric survival models for K-M curve of PFS2 in patients with Nivolumab plus ipilimumab,(B) Survival plots for goodness-of-fit of parametric survival models for K-M curve of PFS2 in patients with Lenvatinib or sorafenib.

# **Figure S6 Scatter plots for probabilistic sensitivity analysis.**


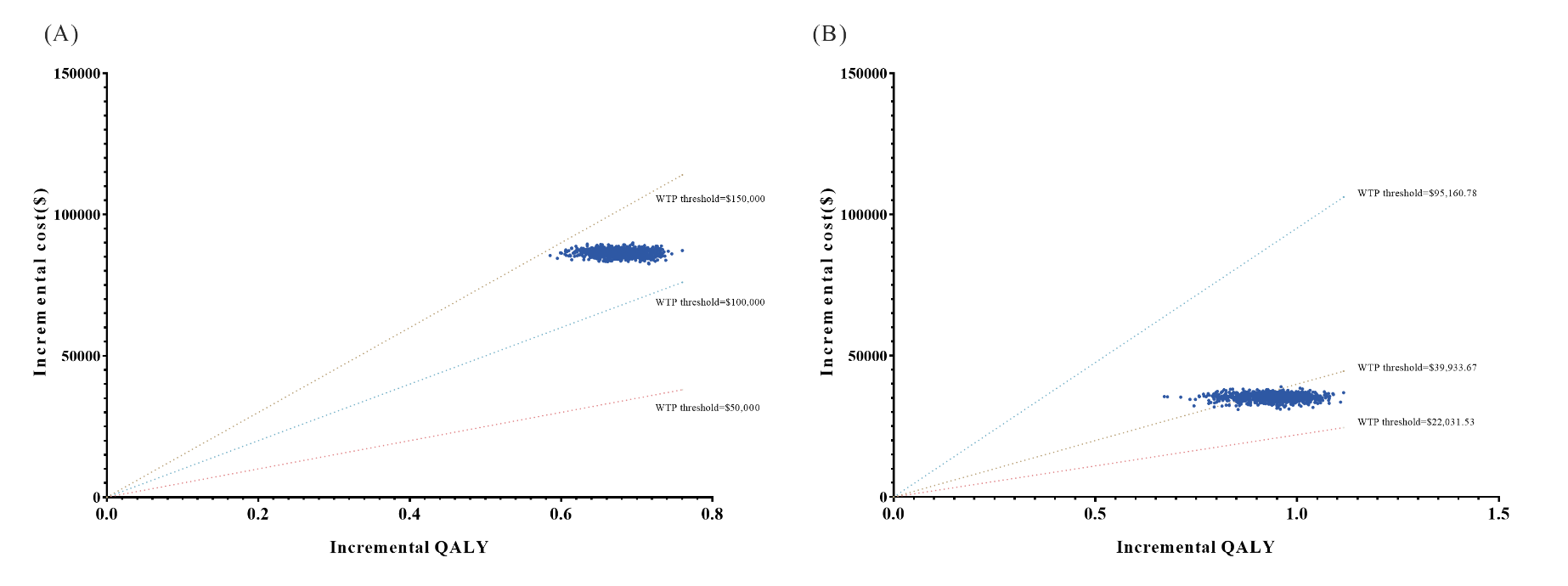


Scatter plots for Nivolumab plus ipilimumab strategy compared with lenvatinib or sorafenib strategy in the United States (A) and China (B).

# **Figure S7 Two-way Sensitivity analysis of Nivolumab plus ipilimumab**


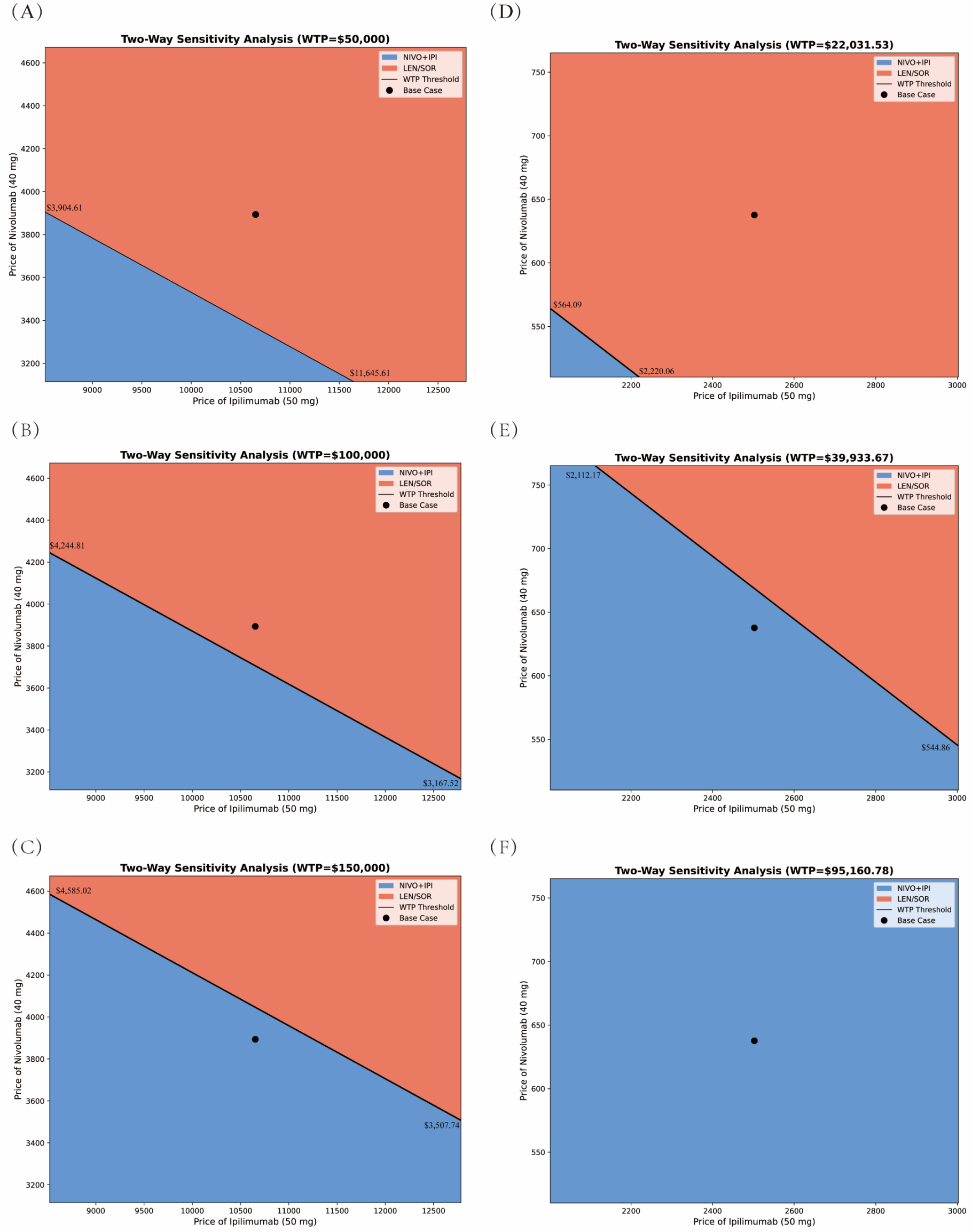


(A) Two-way sensitivity analysis of Nivolumab plus ipilimumab in US ,WTP =$50,000; (B) Two-way sensitivity analysis of Nivolumab plus ipilimumab in US ,WTP =$100,000; (C) Two-way sensitivity analysis of Nivolumab plus ipilimumab in US ,WTP =$150,000; (D) Two-way sensitivity analysis of Nivolumab plus ipilimumab in China ,WTP =$ 22,031.53; (E) Two-way sensitivity analysis of Nivolumab plus ipilimumab in China,WTP =$ 39,933.67; (F) Two-way sensitivity analysis of Nivolumab plus ipilimumab in China,WTP =$ 95,160.67;

# **Table S1 CHEERS 2022 Checklist**

|  | **Item** | **Guidance for Reporting** | **Reported in section** |
| --- | --- | --- | --- |
| **TITLE** | | |  |
| Title | 1 | Identify the study as an economic evaluation and specify the interventions being compared. | √ |
| **ABSTRACT** | | |  |
| Abstract | 2 | Provide a structured summary that highlights context, key methods, results and alternative analyses. | √ |
| **INTRODUCTION** | | |  |
| Background and objectives | 3 | Give the context for the study, the study question and its practical relevance for decision making in policy or practice. | √ |
| **METHODS** | | |  |
| Health economic  analysis plan | 4 | Indicate whether a health economic analysis plan was developed and  where available. |  |
| Study population | 5 | Describe characteristics of the study population (such as age range, demographics, socioeconomic, or clinical characteristics). | √ |
| Setting and location | 6 | Provide relevant contextual information that may influence findings. |  |
| Comparators | 7 | Describe the interventions or strategies being compared and why chosen. | √ |
| Perspective | 8 | State the perspective(s) adopted by the study and why chosen. | √ |
| Time horizon | 9 | State the time horizon for the study and why appropriate. | √ |
| Discount rate | 10 | Report the discount rate(s) and reason chosen. | √ |
| Selection of outcomes | 11 | Describe what outcomes were used as the measure(s) of benefit(s) and harm(s). | √ |
| Measurement of outcomes | 12 | Describe how outcomes used to capture benefit(s) and harm(s) were measured. | √ |
| Valuation of outcomes | 13 | Describe the population and methods used to measure and value outcomes. | √ |
| Measurement and valuation of resources  and costs | 14 | Describe how costs were valued. | √ |
| Currency, price date, and conversion | 15 | Report the dates of the estimated resource quantities and unit costs, plus the currency and year of conversion. | √ |
| Rationale and  description of model | 16 | If modelling is used, describe in detail and why used. Report if the model  is publicly available and where it can be accessed. | √ |
| Analytics and assumptions | 17 | Describe any methods for analysing or statistically transforming data, any extrapolation methods, and approaches for validating any model used. | √ |
| Characterizing heterogeneity | 18 | Describe any methods used for estimating how the results of the study vary for sub-groups. | √ |
| Characterizing  distributional effects | 19 | Describe how impacts are distributed across different individuals  or adjustments made to reflect priority populations. | √ |
| Characterizing uncertainty | 20 | Describe methods to characterize any sources of uncertainty in the analysis. | √ |
| Approach to engagement with patients and others affected by the study | 21 | Describe any approaches to engage patients or service recipients, the general public, communities, or stakeholders (e.g., clinicians or payers) in the design of the study. | √ |
| **RESULTS** | | |  |
| Study parameters | 22 | Report all analytic inputs (e.g., values, ranges, references) including uncertainty or distributional assumptions. | √ |
| Summary of main results | 23 | Report the mean values for the main categories of costs and outcomes of interest and summarise them in the most appropriate overall measure. | √ |
| Effect of uncertainty | 24 | Describe how uncertainty about analytic judgments, inputs, or projections  affect findings. Report the effect of choice of discount rate and time horizon, if applicable. | √ |
| Effect of engagement with patients and others affected by the study | 25 | Report on any difference patient/service recipient, general public, community, or stakeholder involvement made to the approach or findings of the study | √ |
| **DISCUSSION** | | |  |
| Study findings, limitations, generalizability, and current knowledge | 26 | Report key findings, limitations, ethical or equity considerations not captured, and how these could impact patients, policy, or practice. | √ |
|  | | | |
| Source of funding | 27 | Describe how the study was funded and any role of the funder in the identification, design, conduct, and reporting of the analysis | √ |
| Conflicts of interest | 28 | Report authors conflicts of interest according to journal or  International Committee of Medical Journal Editors requirements. | √ |

# **Table S2 Model choice for all survival data**

| **Parameters** | **AIC** | **BIC** | **Value** |
| --- | --- | --- | --- |
| **Nivolumab plus ipilimumab-OS, US** | | | |
| Exponential | 1737.861 | 1741.675 | Rate=0.027537 |
| Gamma | 1739.696 | 1747.324 | Shape=0.9659190, rate=0.0262135 |
| Gompertz | 1736.215 | 1743.844 | Shape=-0.0137500, rate=0.0333042 |
| Weibull | 1739.249 | 1746.877 | Shape=0.951668, scale =36.943408 |
| log-Logistic | 1733.128 | 1740.756 | Shape=1.16393, scale =23.92591 |
| log-Normal | 1727.031 | 1734.659 | Meanlog=3.17526, sdlog =1.47177 |
| Generalised Gamma | 1728.706 | 1740.148 | Mu=3.075627, sigma=1.526658, Q=-0.187073 |
| **Lenvatinib or sorafenib-OS, US** | | | |
| Exponential | 1951.063 | 1954.871 | Rate=0.0350638 |
| Gamma | 1934.891 | 1942.507 | Shape=1.447789, rate=0.055292 |
| Gompertz | 1942.985 | 1950.601 | Shape=0.0207205, rate=0.0260584 |
| Weibull | 1935.693 | 1943.309 | Shape=1.28325, scale =27.61148 |
| log-Logistic | 1938.090 | 1945.706 | Shape=1.64134, scale =19.74340 |
| log-Normal | 1949.447 | 1957.063 | Meanlog=2.96063, sdlog =1.10570 |
| Generalised Gamma | 1936.853 | 1948.277 | Mu=3.251772, sigma=0.845758, Q=0.789452 |
| **Nivolumab plus ipilimumab-OS, China** | | | |
| Exponential | 493.663 | 496.248 | Rate=0.0286547 |
| Gamma | 495.657 | 500.827 | Shape=1.0124746, rate=0.0291673 |
| Gompertz | 495.524 | 500.694 | Shape=-0.00527616, rate=0.03069625 |
| Weibull | 495.658 | 500.828 | Shape=0.991875, scale =35.005551 |
| log-Logistic | 495.072 | 500.242 | Shape=1.19072, scale =23.45711 |
| log-Normal | 492.867 | 498.037 | Meanlog=3.14747, sdlog =1.43376 |
| Generalised Gamma | 494.739 | 502.494 | Mu=3.014272, sigma=1.504301, Q=-0.256575 |
| **Lenvatinib or sorafenib-OS, China** | | | |
| Exponential | 572.120 | 574.820 | Rate=0.0338595 |
| Gamma | 568.374 | 573.775 | Shape=1.4664288, rate=0.0559714 |
| Gompertz | 573.326 | 578.727 | Shape=0.0114036, rate=0.0293362 |
| Weibull | 569.570 | 574.971 | Shape=1.26393, scale =27.78563 |
| log-Logistic | 565.845 | 571.246 | Shape=1.6398, scale =19.5648 |
| log-Normal | 567.661 | 573.062 | Meanlog=2.98383, sdlog =1.09237 |
| Generalised Gamma | 568.745 | 576.847 | Mu=3.112256, sigma=0.986552, Q=0.352973 |
| **Nivolumab plus ipilimumab-PFS** | | | |
| Exponential | 1667.702 | 1671.516 | Rate =0.0606296 |
| Gamma | 1664.569 | 1672.197 | Shape=0.8408727, rate=0.0484306 |
| Gompertz | 1632.381 | 1640.009 | Shape=-0.0535913, rate= 0.0974486 |
| Weibull | 1658.612 | 1666.240 | Shape=0.840556, scale =16.576187 |
| log-Logistic | 1625.916 | 1633.544 | Shape=1.17973, scale =8.86248 |
| log-Normal | 1613.359 | 1620.988 | Meanlog =2.22550, sdlog =1.39973 |
| Generalised Gamma | 1600.199 | 1611.641 | Mu=1.708278, sigma=1.399573, Q=-0.842527 |
| **Lenvatinib or sorafenib-PFS** | | | |
| Exponential | 1548.460 | 1552.268 | rate=0.0770522 |
| Gamma | 1528.813 | 1536.429 | Shape=1.486307, rate=0.123892 |
| Gompertz | 1545.800 | 1553.416 | Shape=0.0212460, rate= 0.0653605 |
| Weibull | 1533.481 | 1541.097 | Shape=1.25461, scale =12.87742 |
| log-Logistic | 1528.431 | 1536.047 | Shape=1.71588, scale =8.71920 |
| log-Normal | 1520.095 | 1527.711 | Meanlog =2.143763, sdlog =0.990959 |
| Generalised Gamma | 1521.867 | 1533.291 | Mu=2.194136, sigma=0.974415, Q=0.116974 |
| **Nivolumab plus ipilimumab-PFS2** | | | |
| Exponential | 1813.060 | 1816.874 | Rate=0.0321008 |
| Gamma | 1815.055 | 1822.683 | Shape=1.006306, rate=0.032376 |
| Gompertz | 1808.949 | 1816.578 | Shape=-0.0178367, rate= 0.0404775 |
| Weibull | 1814.803 | 1822.432 | Shape=0.969885, scale =31.386514 |
| log-Logistic | 1801.899 | 1809.527 | Shape=1.23096, scale =19.81493 |
| log-Normal | 1793.563 | 1801.191 | Meanlog =2.99101, sdlog =1.37053 |
| Generalised Gamma | 1792.588 | 1804.030 | Mu=2.696772, sigma=1.455761, Q=-0.533335 |
| **Lenvatinib or sorafenib-PFS2** | | | |
| Exponential | 2073.446 | 2077.254 | rate=0.0460674 |
| Gamma | 2040.919 | 2048.535 | Shape=1.6486603, rate=0.0817571 |
| Gompertz | 2065.627 | 2073.243 | Shape=0.0197887, rate= 0.0356788 |
| Weibull | 2046.888 | 2054.504 | Shape=1. 33923, scale =21.77252 |
| log-Logistic | 2035.173 | 2042.790 | Shape=1.86155, scale =15.23081 |
| log-Normal | 2038.653 | 2046.270 | Meanlog =2.707596, sdlog =0.943117 |
| Generalised Gamma | 2037.457 | 2048.882 | Mu=2.840376, sigma=0.882392, Q=0.337839 |

AIC Akaike Information Criterion ,BIC Bayesian Information Criterion,OS overall survival, PFS progression-free survival.

# **Table S3 Drug acquisition unit costs in the model**

| **Drugs used in the model** | **Method and frequency of administration** | **Dose per vial/pack** | **Cost per vial/pack ($)-US** | | **Source** | **Cost per vial/pack ($)-CN** | | **Source** |
| --- | --- | --- | --- | --- | --- | --- | --- | --- |
|  |  |  | Mean | SD |  | Mean | SD |  |
| **Nivolumab** | 1mg/kg q3w cycle 4 followed 480 mg q4w | 10mg*4ml | 3,893.65 | NA | ^1^ | 637.66 | NA | ^2^ |
| **Ipilimumab** | 3mg/kg q3w cycle 4 | 5mg*10ml | 10,652.64 | NA | ^1^ | 2,502.40 | NA | ^2^ |
| **Lenvatinib** | 8/12mg po qd | 4mg*90 | 29,979.60 | NA | ^1^ | 172.63 | 133.93 | ^2^ |
| **Sorafenib** | 400mg bid qd | 200 mg*120 | 25,299.00 | NA | ^1^ | 247.10 | 281.98 | ^2^ |
| **Bevacizumab** | 15mg/kg q3w | 25 ml*4ml | 956.33 | NA | ^1^ | 158.74 | 17.29 | ^2^ |
| **Atezolizumab** | 1200mg q3w | 60 mg*20ml | 13,502.17 | NA | ^1^ | 4,559.93 | NA | ^2^ |
| **Regorafenib** | 160mg d1-d21, q4w | 40mg*28 | 9,537.36 | NA | ^1^ | 279.73 | 253.40 | ^2^ |
| **Cabozantinib** | 60mg po qd | 60mg*30 | 31.561.43 | NA | ^1^ | NA | NA | NA |
| **Apatinib** | 750mg po qd | 0.25g*10 | NA | NA | NA | 145.53 | NA | ^2^ |

# **Table S4 Adverse events occurrences, unit costs of AE management.**

| **Adverse events** | **Incidence (O+I)** | **Incidence (L/S)** | **US-Cost/$** | **Source** | **CN-Cost/$** | **Source** |
| --- | --- | --- | --- | --- | --- | --- |
| **ALT increased** | 5% | 0.9% | 43,801.24 | HCUP(SYM017) ^3,4^ | 89.5 | ^5,6^ |
| **AST increased** | 6% | 0.6% | 43,801.24 | HCUP(SYM017) ^3,4^ | 89.5 | ^5,6^ |
| **PPE syndrome** | 0 | 3% | 19,733.06 | HCUP(SKN002) ^3,4^ | 1,112.69 | Estimated |
| **Hypertension** | 0 | 12 % | 175,836.04 | HCUP(CIR008) ^3,4^ | 655.27 | ^6,7^ |
| **Proteinuria** | 0 | 5% | 104.26 | HCUP(GEN010) ^3,4^ | 356.04 | ^6,8^ |

N+I Nivolumab plus ipilimumab, L/S Lenvatinib or sorafenib. AE Adverse events

# **Table S5 Subsequent anti-cancer therapies for patients in the Nivolumab plus ipilimumab and Lenvatinib or sorafenib groups**

| **All randomised** | **Nivolumab plus ipilimumab**  **(n=335)** | **Lenvatinib or sorafenib**  **(n=333)** |
| --- | --- | --- |
| **Any subsequent therapy^*^** | 151 (45%) | 185 (56%) |
| **Subsequent radiotherapy** | 21 (6%) | 25 (8%) |
| **Subsequent surgery** | 12 (4%) | 6 (2%) |
| **Subsequent locoregional therapy** | 29 (9%) | 7 (8%) |
| **Subsequent systemic therapy** | 128 (38%) | 172 (52%) |
| Any immunotherapy | 44 (13%) | 115 (35%) |
| Any immunotherapy-containing combination regimen | 36 (11%) | 78 (23%) |
| Anti-VEGF agents | 95 (28%) | 99 (30%) |
| Other | 6 (2%) | 11 (3%) |

**For patients received no subsequent anti-cancer therapies (55% in the Nivolumab plus ipilimumab and 44% in the Lenvatinib or sorafenib group), best supportive care was used.

# **Table S6 Variables applied in the economic model**

| **Healthcare resource** | **US** | **Reference** | **CN** | **Reference** |
| --- | --- | --- | --- | --- |
| All iv therapies* | **$**144.09 | ^9^ | **$**143.24 | Estimated |
| Laboratory/time | **$**24.30 | ^3,10^ | **$**26.02 | Estimated |
| abdomen CT | **$**351 | ^11^ | **$**176.77 | Estimated |
| Thorax CT | **$**184 | ^11^ | **$**57.03 | Estimated |
| Brain MRI | $270 | ^11^ | **$**143.25 | Estimated |
| Cost of best supportive | **$**794.28 | ^3,12^ | **$**376.69 | ^6,13^ |
| End of life cost | **$**11,762.07 | ^3,12^ | **$**8,903.81 | ^6,14^ |
| Discount for cost and utility | 0.03(0-0.08) | ^15^ | 0.05(0-0.08) | ^16^ |
| Weight(kg) |  |  |  |  |
| Man | 90.63 | ^17^ | 68.1 | ^18^ |
| Woman | 77.47 | ^17^ | 59.8 | ^18^ |
| Height(cm) |  |  |  |  |
| Man | 175.26 cm | ^17^ | 165.4 | ^18^ |
| Woman | 161.29cm | ^17^ | 154.4 | ^18^ |

# **Table S7 Health state utility values**

| **Parameter** | **Value** | **SD** | **Distribution** | **Source** |
| --- | --- | --- | --- | --- |
| PFS | 0.84 | 0.02274 | beta | ^19,20^ |
| PFS2 | 0.76 | 0.08376 | beta | ^19,21^ |
| PD | 0.68 | 0.07157 | beta | ^22^ |

# **Table S8 Disutility of AE**

| **Disutility** | Value | Distribution | Source |
| --- | --- | --- | --- |
| **ALT increased** | 0.05 | beta | ^23^ |
| **AST increased** | 0.05 | beta | ^23^ |
| **PPE syndrome** | 0.10 | beta | ^24^ |
| **Hypertension** | 0.12 | beta | ^25^ |
| **Proteinuria** | 0.048 | beta | ^26^ |

AE Adverse events

# **Table S9 Scenario analysis results: Cost-effectiveness of Nivolumab plus Ipilimumab versus Lenvatinib or Sorafenib monotherapy**

| **Strategy** | **US** | | | | | **CN** | | | | |
| --- | --- | --- | --- | --- | --- | --- | --- | --- | --- | --- |
|  | **Costs ($)** | **Incremental**  **costs ($)** | **Effectiveness**  **(QALY)** | **Incremental**  **effectiveness** | **ICER ($)** | **Costs ($)** | **Incremental**  **costs ($)** | **Effectiveness**  **(QALY)** | **Incremental**  **effectiveness** | **ICER ($)** |
| **Lenvatinib** | |  |  |  |  |  |  |  |  |  |
| NIVO+IPI | 890,996.08 | 348,190.63 | 3.73 | 1.72 | 201,266.26 | 106,885.04 | 73,507.70 | 3.73 | 1.72 | 42,641.28 |
| Lenvatinib | 542,805.46 |  | 2.01 |  |  | 33,378.34 |  | 2.01 |  |  |
| **Sorafenib** | |  |  |  |  |  |  |  |  |  |
| NIVO+IPI | 989,046.29 | 529,650.94 | 4.44 | 2.94 | 179,934.87 | 101,840.11 | 82,373.43 | 4.44 | 2.94 | 27,984.19 |
| sorafenib | 459,395.35 |  | 1.50 |  |  | 19,466.68 |  | 1.50 |  |  |

NIVO+IPI Nivolumab plus ipilimumab, LEN/SOR lenvatinib or sorafenib,QALY quality adjusted life-year, ICER incremental cost-effectiveness ratio, US United States

# **Table S10 Scenario analysis applying overall survival data from the global population to the Chinese setting**

| **Strategy** | **Costs ($)** | **Incremental**  **costs ($)** | **Effectiveness**  **(QALY)** | **Incremental**  **effectiveness** | **ICER ($)** |
| --- | --- | --- | --- | --- | --- |
| **All Patients** | |  |  |  |  |
| NIVO+IPI | 106,489.73 | 30,980.87 | 3.20 | 0.68 | 45,560.10 |
| LEN/SOR | 75,508.86 |  | 2.52 |  |  |
| **Only PFS** | |  |  |  |  |
| NIVO+IPI | 98,541.59 | 80,285.69 | 1.26 | 0.39 | 205,860.74 |
| LEN/SOR | 18,255.90 |  | 0.87 |  |  |
| **PFS investigator** | |  |  |  |  |
| NIVO+IPI | 107,314.46 | 31,701.58 | 3.86 | 1.39 | 22,865.84 |
| LEN/SOR | 75,612.88 |  | 2.47 |  |  |
| **Exclude AE disutilities** | |  |  |  |  |
| NIVO+IPI | 30,980.86 | 3.22 | 0.69 | 44,729.89 | 30,980.86 |
| LEN/SOR |  | 2.53 |  |  |  |
| **time horizon 4 years** | | |  |  |  |
| NIVO+IPI | 105,309.75 | 42,516.00 | 2.22 | 0.2 | 212,579.99 |
| LEN/SOR | 62,793.75 |  | 2.02 |  |  |
| **time horizon 5 years** | | |  |  |  |
| NIVO+IPI | 106,062.72 | 38,860.16 | 2.49 | 0.3 | 129,533.87 |
| LEN/SOR | 67,202.56 |  | 2.19 |  |  |
| **time horizon 10 years** | | |  |  |  |
| NIVO+IPI | 106,486.66 | 32,257.96 | 3.05 | 0.61 | 106,486.66 |
| LEN/SOR | 74,228.70 |  | 2.44 |  | 74,228.70 |

NIVO+IPI Nivolumab plus ipilimumab, QALY quality adjusted life-year, ICER incremental cost-effectiveness ratio, US United States

# References

1. "Merative Micromedex ®RED BOOK®.", <https://www.micromedexsolutions.com/micromedex2/librarian/CS/DBBB46/ND_PR/evidencexpert/ND_P/evidencexpert/DUPLICATIONSHIELDSYNC/454140/ND_PG/evidencexpert/ND_B/evidencexpert/ND_AppProduct/evidencexpert/ND_T/evidencexpert/PFActionId/redbook.FindRedBook?navitem=topRedBook&isToolPage=true>, accessed May 20, 2025.

2. "DrugDataexpy." <https://data.yaozh.com/>, accessed May 20, 2025.

3. "Consumer price index. United States Bureau of Labor Statistics.", <https://www.bls.gov/cpi/>. , accessed May 20, 2025.

4. "The Healthcare Cost and Utilization Project (HCUP) ", <https://datatools.ahrq.gov/hcupnet/>, accessed May 20, 2025.

5. Wen F, Zheng H, Zhang P, et al: Atezolizumab and bevacizumab combination compared with sorafenib as the first-line systemic treatment for patients with unresectable hepatocellular carcinoma: A cost-effectiveness analysis in China and the United states. Liver Int 41:1097-1104, 2021. <https://doi.org/10.1111/liv.14795>.

6. "National Bureau of Statistice.National data." <https://data.stats.gov.cn/english/easyquery.htm?cn=C01>, accessed May 20, 2025.

7. Xie X, He T, Kang J, et al: Cost-effectiveness analysis of intensive hypertension control in China. Prev Med 111:110-114, 2018. <https://doi.org/10.1016/j.ypmed.2018.02.033>.

8. Wen F, Wang J, Yang C, et al: Cost-effectiveness of population-based screening for chronic kidney disease among the general population and adults with diabetes in China: a modelling study. Lancet Reg Health West Pac 56:101493, 2025. <https://doi.org/10.1016/j.lanwpc.2025.101493>.

9. "CMS.gov.Physician Fee Schedule.", <https://www.cms.gov/medicare/physician-fee-schedule/search?Y=3&T=0&HT=0&CT=3&H1=80076&M=5>, accessed May 20, 2025.

10. Shao T, Zhao M, Liang L, Tang W: Serplulimab Plus Chemotherapy vs Chemotherapy for Treatment of US and Chinese Patients with Extensive-Stage Small-Cell Lung Cancer: A Cost-Effectiveness Analysis to Inform Drug Pricing. BioDrugs 37:421-432, 2023. <https://doi.org/10.1007/s40259-023-00586-6>.

11. "Medicare.gov." <https://www.medicare.gov/procedure-price-lookup/>, accessed May 20, 2025.

12. Criss SD, Mooradian MJ, Watson TR, et al: Cost-effectiveness of Atezolizumab Combination Therapy for First-Line Treatment of Metastatic Nonsquamous Non-Small Cell Lung Cancer in the United States. JAMA Netw Open 2:e1911952, 2019. <https://doi.org/10.1001/jamanetworkopen.2019.11952>.

13. Gu X, Zhang Q, Chu Y, et al: Cost-effectiveness of afatinib, gefitinib, erlotinib and pemetrexed-based chemotherapy as first-line treatments for advanced non-small cell lung cancer in China. 127:84-89, 2019. <https://doi.org/10.1016/j.lungcan.2018.11.029>.

14. Zeng X, Karnon J, Wang S, et al: The cost of treating advanced non-small cell lung cancer: estimates from the chinese experience. PLoS One 7:e48323, 2012. <https://doi.org/10.1371/journal.pone.0048323>.

15. Neumann PJ, Cohen JT, Weinstein MC: Updating cost-effectiveness--the curious resilience of the $50,000-per-QALY threshold. N Engl J Med 371:796-7, 2014. <https://doi.org/10.1056/NEJMp1405158>.

16. Guoen L. Chinese Guidelines for Pharmacoeconomics Evaluation 2020. China Market Press; 2020

17. "Body Measurements.", <https://www.cdc.gov/nchs/fastats/body-measurements.htm>, accessed May 20, 2025.

18. "General Administration of Sport of China. Fifth National physical fitness monitoring bulletin (2022). ." <https://www.sport.gov.cn/n315/n329/c24335066/content.html>, accessed May 20, 2025.

19. Chiang CL, Chan SK, Lee SF, et al: Cost-effectiveness of Pembrolizumab as a Second-Line Therapy for Hepatocellular Carcinoma. JAMA Netw Open 4:e2033761, 2021. <https://doi.org/10.1001/jamanetworkopen.2020.33761>.

20. Zhu AX, Finn RS, Edeline J, et al: Pembrolizumab in patients with advanced hepatocellular carcinoma previously treated with sorafenib (KEYNOTE-224): a non-randomised, open-label phase 2 trial. Lancet Oncol 19:940-952, 2018. <https://doi.org/10.1016/s1470-2045(18)30351-6>.

21. Soto-Perez-de-Celis E, Aguiar PN, Cordón ML, et al: Cost-Effectiveness of Cabozantinib in the Second-Line Treatment of Advanced Hepatocellular Carcinoma. J Natl Compr Canc Netw 17:669-675, 2019. <https://doi.org/10.6004/jnccn.2018.7275>.

22. Cammà C, Cabibbo G, Petta S, et al: Cost-effectiveness of sorafenib treatment in field practice for patients with hepatocellular carcinoma. Hepatology 57:1046-54, 2013. <https://doi.org/10.1002/hep.26221>.

23. Batteson R, Hook E, Wheat H, et al: Modelling the Effectiveness of Tepotinib in Comparison to Standard-of-Care Treatments in Patients with Advanced Non-small Cell Lung Cancer (NSCLC) Harbouring METex14 Skipping in the UK. Target Oncol 19:191-201, 2024. <https://doi.org/10.1007/s11523-024-01038-z>.

24. Nafees B, Lloyd AJ, Dewilde S, et al: Health state utilities in non-small cell lung cancer: An international study. Asia Pac J Clin Oncol 13:e195-e203, 2017. <https://doi.org/10.1111/ajco.12477>.

25. Saiyed M, Byrnes J, Srivastava T, et al: Cost-Effectiveness of Lenvatinib Compared with Sorafenib for the First-Line Treatment of Advanced Hepatocellular Carcinoma in Australia. Clin Drug Investig 40:1167-1176, 2020. <https://doi.org/10.1007/s40261-020-00983-7>.

26. Beaudet A, Clegg J, Thuresson PO, et al: Review of utility values for economic modeling in type 2 diabetes. Value Health 17:462-70, 2014. <https://doi.org/10.1016/j.jval.2014.03.003>.
